# Risk factors for coronary artery abnormalities and resistance to immunoglobulin plus ciclosporin A therapy in severe Kawasaki disease: subanalysis of the KAICA trial, randomized trial for cicrosporin A as the first-line treatment

**DOI:** 10.3389/fped.2023.1321533

**Published:** 2023-12-15

**Authors:** Yuri Murayama, Hiromichi Hamada, Yuki Shiko, Yoshihiro Onouchi, Nobuyuki Kakimoto, Yoshihito Ozawa, Hideki Hanaoka, Akira Hata, Hiroyuki Suzuki

**Affiliations:** ^1^ Department of Pediatrics, School of Medicine, Wakayama Medical University, Wakayama, Japan; ^2^ Department of Pediatrics, Graduate School of Medicine, Chiba University, Chiba, Japan; ^3^ Clinical Research Centre, Chiba University Hospital, Chiba, Japan; ^4^ Department of Public Health, Graduate School of Medicine, Chiba University, Chiba, Japan; ^5^ Department of Health Research, Chiba Foundation for Health Promotion and Disease Prevention, Chiba, Japan; ^6^ Division of Pediatrics, Tsukushi Medical and Welfare Center, Iwade, Japan

**Keywords:** Kawasaki disease, coronary artery disease, immunoglobulin, ciclosporin A, subanalysis

## Abstract

**Background:**

To investigate risk factors for coronary arterial abnormalities (CAAs) and resistance to treatment in patients with Kawasaki disease (KD) receiving intravenous immunoglobulin (IVIG) plus ciclosporin A (CsA) as the first-line treatment, we performed a subanalysis of baseline data of participants in the KAICA trial, a phase 3, randomized study (JMA-ILA00174).

**Methods:**

All data of the patients enrolled in the KAICA trial, who had a Gunma score ≥5 at diagnosis and had been randomly assigned to either IVIG (2 g/kg/24 h) plus CsA (5 mg/kg/day for 5 days) (*n* = 86) or IVIG alone (*n* = 87), were subjected to this study. CAA was defined by a *Z* score ≥2.5 observed within 4 weeks after treatment initiation. Baseline data including genotypes of KD susceptibility genes were compared between subgroups of patients for CAA or treatment response for each treatment group. Backword-forward stepwise logistic regression analyses were performed.

**Results:**

Pre-Z-max, defined as the maximum among *Z* scores on four coronary artery branches before treatment, was higher in patients with CAA in both treatment groups and was associated with CAA in IVIG plus CsA treatment group [odds ratio (OR) = 17.0]. High serum total bilirubin level was relevant to treatment resistance only in the IVIG plus CsA group (OR = 2.34).

**Conclusions:**

Coronary artery enlargement before treatment is a major determinant of CAA even in KD patients treated with initial IVIG treatment intensified by addition of CsA. Baseline serum total bilirubin level was a risk factor associated with resistance to IVIG plus CsA.

## 1. Introduction

Kawasaki disease (KD) is an acute systemic vasculitis of childhood that can cause dilation or aneurysmal change in the coronary arteries in 20%–25% of untreated patients (1). Coronary artery abnormalities (CAAs) in KD are now the leading cause of acquired heart diseases in developed countries (2). The aim of the acute phase treatment of KD is to prevent CAAs (3). Proven effective in the 1980s for the early resolution of inflammation and prevention of CAA development, the combination of intravenous immunoglobulin (IVIG) and oral aspirin is now the standard first-line treatment given to most patients with KD. However, resistance to this treatment is seen in around 20% of KD patients and is recognized as one of the major risk factors for CAAs.

CAAs that cannot be prevented by adequate administration of the first-line treatment have become a major clinical issue (3, 4). Recently, in an attempt to develop intensified treatment regimens that can more efficiently prevent CAAs, a series of prospective randomized clinical trials of drugs administered as adjuncts to the standard IVIG treatment has been carried out (5–7). Ciclosporin A (CsA), an inhibitor of the calcineurin-NFAT pathway, is one such drug. It was shown in our previous clinical trial (the KAICA trial, a controlled, phase 3, randomised, open-label, blinded-endpoints trial) to reduce the incidence of CAAs when administered to KD patients with high predictive scores for IVIG unresponsiveness (Gunma score) in combination with the standard IVIG therapy (5, 8–10). Thus, there are now four options that can be combined with the standard IVIG therapy for treating severe KD patients in the Japanese treatment guidelines, two that are recommended (prednisolone and CsA) and two that can be considered (infliximab and ulinastatin) (3, 11). The efficacy of IL-1 blocking therapies has been initiated in Europe and US (12, 13). However, none of the intensified regimens can eradicate CAAs. Although the incidence of CAAs was significantly reduced when compared with those receiving the conventional IVIG treatment (31%), 14% of the patients receiving IVIG plus CsA in the KAICA trial still had CAAs (5).

To investigate risk factors for CAAs and resistance to IVIG plus CsA as the first-line treatment in patients with KD, we performed a subanalysis of baseline data of participants in the KAICA trial.

## 2. Methods

### 2.1. Patients

This study was performed as a subanalysis of the KAICA trial (JMA-ILA00174, date on registration: April 2, 2014), which was a phase 3, randomized, open-label, blinded endpoint study (5). In this clinical study, 173 patients who were diagnosed with KD according to Japanese diagnostic guidelines and predicted to be non-responders to IVIG by Gunma score were randomly assigned 1:1 to IVIG plus CsA or IVIG alone (5, 10, 14). There were 86 patients in the study treatment group who received CsA 5 mg/kg per day orally divided into two daily doses for 5 days in addition to IVIG and aspirin and 87 patients in the conventional treatment group who received IVIG 2 g/kg for 24 h and aspirin 30 mg/kg per day. These 173 patients were enrolled in the present analysis.

### 2.2. Definition of CAAs

In the KAICA trial, absolute internal diameters of the right coronary artery, left main coronary artery, left anterior descending coronary artery, and left circumflex coronary artery were measured by three pediatric cardiologists using video-recorded data at six time points—once before the first-line treatment and at five time points after treatment initiation, namely, on day 3 and at week 1, week 2, week 4, and week 12—without any clinical information and centrally reviewed. In this subanalysis, we did not include the week 12 data in this study.

Coronary artery *Z* scores were calculated using the *Z* scores calculator (version 4.0 full, LMS_*Z*_Score) (15). A *Z* score of 2.5 was set as the threshold for the diagnosis of coronary artery dilation according to the 2017 American Heart Association guidelines (3). Patients with one or more *Z* scores ≥2.5 at any time point up to 4 weeks after treatment initiation were defined as having a CAA in this study. The maximum *Z* score of the four branches measured and calculated before the first-line treatment was defined as the “pre-Z-max”.

### 2.3. Definition of response to the first-line treatment

In both treatment groups, patients whose body temperature dropped below 37.5°C within 48 h after the initiation of the first-line treatment and who remained afebrile thereafter were defined as the responders to the first-line treatment. The other patients, including those with persistent fever beyond 48 h after the initiation of the first-line treatment or those becoming febrile again after an afebrile period during the 48 h, were defined to be resistant to the first-line treatment.

### 2.4. Baseline data and analysis plan

We extracted data of registered patients regarding basic characteristics from the KAICA trial database, such as height, weight, age, days of illness at diagnosis, days of illness at treatment initiation, single-nucleotide polymorphisms (SNPs) in *inositol 1,4,5-trisphosphate 3-kinase C* (*ITPKC*; rs28493229) and *caspase-3* (*CASP3*; rs113420705), family history of KD, vital signs (blood pressure, heart rate, body temperature), baseline clinical data before any treatments (white blood cell [WBC] counts, WBC analysis [%], red blood cell [RBC] count, hemoglobin [Hgb], hematocrit [Hct], platelet count, aspartate aminotransferase [AST], alanine aminotransferase [ALT], total bilirubin [TB], total protein [TP], albumin [Alb], sodium [Na], potassium [K], magnesium [Mg], blood urea nitrogen [BUN], creatinine [Cre], C-reactive protein [CRP], and pre-Z-max).

Data were compared separately between patients with and without CAAs in the two treatment groups. Of the 87 patients in the IVIG group, 2 had no pre-Z-max data, so the analysis regarding CAAs was performed on 85 patients in the IVIG group.

We compared data between patients with and without a treatment response for 86 patients who received IVIG plus CsA. We also extracted the baseline data of 87 registered patients treated with IVIG alone and compared these data between patients with and without a treatment response.

### 2.5. Statistical analysis

Categorical variables are expressed as absolute numbers and percentages and were compared using Fisher's exact test, whereas continuous variables are expressed as mean and standard deviation and were compared using Student's *t*-test. Multivariate logistic regression analysis with backward-forward stepwise variable selection method was performed to identify the independent variables associated with CAAs or treatment resistance. All statistical analyses were performed using the SAS statistical software package (version 9.4; SAS Institute), with *p*-values < 0.05 indicating statistical significance.

This study is approved by Chiba University Ethical committee review board (No. 843).

## 3. Results

### 3.1. Risk factors for CAA

The baseline data of the two treatment groups in the KAICA trial are shown in Table 1. In both treatment groups, 19 patients had CAAs defined by a *Z* score ≥2.5, and the mean pre-Z-max was significantly higher in those with CAA (*p* < 0.0001). In the IVIG plus CsA group, baseline RBC, Hgb, and Hct levels were significantly lower in the patients with CAAs (*p* = 0.004, 0.041, and 0.028, respectively). For these hematological markers related to RBCs, a similar trend was seen in the IVIG group; however, only the difference in the Hct was significant (*p* = 0.045). There were no significant differences in the other demographic, physical, hematological, and biochemical data or in the frequencies of risk allele carriers of *ITPKC* and *CASP3* SNPs between those with and without CAAs in either group. Backward-forward stepwise multivariate logistic regression analyses revealed that pre-Z-max was the only significant predicted factor in IVIG + CsA group (Table 2).

**Table 1:** Difference in baseline data between KD patients with or without CAA in IVIG plus CsA and IVIG groups.

	IVIG + CsA group				IVIG group			
	CAA(−) (*N* = 67)	CAA(+) (*N* = 19)	Total (*N* = 86)	*P*-value	CAA(−) (*N* = 66)**	CAA(+) (*N* = 19)	Total (*N* = 85)	*P*-value
Pre-Z-max, mean (SD)	1.4 (0.64)	2.3 (0.96)	1.6 (0.81)	<0.001	1.3 (0.76)	2.1 (0.89)	1.5 (0.86)	<0.001
Age (months), mean (SD)	39.8 (30.41)	30.2 (32.95)	37.7 (31.05)	0.233	35.0 (22.44)	41.3 (27.80)	36.4 (23.71)	0.310
Gunma score, mean (SD)	6.5 (1.46)	6.8 (1.40)	6.5 (1.44)	0.387	6.4 (1.57)	6.5 (1.71)	6.4 (1.59)	0.779
Height (cm), mean (SD)	92.1 (18.13)	83.1 (20.19)	90.1 (18.86)	0.066	89.8 (15.76)	94.5 (19.63)	90.9 (16.69)	0.279
Weight (kg), mean (SD)	13.5 (5.01)	11.5 (5.39)	13.0 (5.13)	0.150	12.8 (4.09)	14.8 (6.82)	13.2 (4.86)	0.108
illness day of starting 1st-IVIG, mean (SD)	4.4 (1.25)	4.4 (0.83)	4.4 (1.17)	0.973	4.3 (1.12)	4.1 (1.13)	4.2 (1.12)	0.452
illness day of diagnosis, mean (SD)	4.2 (1.31)	4.2 (0.85)	4.2 (1.22)	0.996	4.1 (1.17)	3.9 (1.08)	4.1 (1.15)	0.563
Systolic pressure, mean (SD)	101.8 (11.89)	105.1 (11.92)	102.6 (11.90)	0.295	102.6 (12.57)	99.2 (11.53)	101.8 (12.35)	0.291
Diastolic pressure, mean (SD)	57.4 (10.76)	61.4 (12.72)	58.3 (11.28)	0.171	58.4 (13.32)	52.9 (8.28)	57.1 (12.48)	0.094
Heart rate, mean (SD)	145.3 (26.22)	157.2 (27.18)	147.9 (26.74)	0.086	143.4 (20.02)	149.3 (22.18)	144.7 (20.54)	0.269
With/without 3rd line treatment, *n* (%)								
Without	56 (83.6)	15 (78.9)	71 (82.6)	0.733*	56 (84.8)	15 (78.9)	71 (83.5)	0.5045*
With	11 (16.4)	4 (21.1)	15 (17.4)		10 (15.2)	4 (21.1)	14 (16.5)	
Red blood cells (10^6^/μl), mean (SD)	4.4 (0.33)	4.1 (0.30)	4.3 (0.34)	0.004	4.4 (0.39)	4.3 (0.45)	4.4 (0.40)	0.352
Hgb (g/dl), mean (SD)	11.5 (1.14)	10.9 (0.88)	11.4 (1.11)	0.041	11.7 (1.21)	11.1 (1.01)	11.6 (1.19)	0.062
Hct (%), mean (SD)	34.2 (3.11)	32.5 (2.23)	33.8 (3.01)	0.028	34.6 (3.09)	33.1 (2.12)	34.3 (2.96)	0.045
Platelet (10^3^/μl), mean (SD)	280.9 (71.11)	305.5 (100.13)	286.3 (78.46)	0.229	299.9 (85.55)	274.9 (63.83)	294.3 (81.52)	0.242
AST (U/L), mean (SD)	111.9 (96.10)	95.1 (83.66)	108.2 (93.28)	0.493	141.8 (126.68)	167.2 (152.83)	147.5 (132.42)	0.464
ALT (U/L), mean (SD)	115.7 (95.00)	121.2 (131.86)	116.9 (103.41)	0.838	121.3 (107.41)	157.9 (143.53)	129.5 (116.53)	0.229
TB (mg/dl), mean (SD)	1.2 (1.07)	1.5 (1.62)	1.3 (1.21)	0.365	1.1 (1.07)	1.5 (1.28)	1.2 (1.12)	0.248
TP (g/dl), mean (SD)	6.4 (0.64)	6.1 (0.76)	6.3 (0.67)	0.156	6.5 (0.62)	6.4 (0.62)	6.5 (0.62)	0.716
Alb (g/dl), mean (SD)	3.5 (0.39)	3.6 (0.48)	3.6 (0.41)	0.354	3.7 (0.43)	3.6 (0.46)	3.7 (0.44)	0.568
Na (mmol/L), mean (SD)	132.6 (2.77)	132.5 (1.56)	132.6 (2.54)	0.871	132.4 (2.88)	131.6 (2.87)	132.2 (2.88)	0.332
K (mmol/L), mean (SD)	4.2 (0.43)	4.2 (0.57)	4.2 (0.46)	0.957	4.2 (0.42)	4.2 (0.37)	4.2 (0.41)	0.576
Mg (mg/dl), mean (SD)	2.2 (0.23)	2.2 (0.20)	2.2 (0.22)	0.854	2.2 (0.26)	2.2 (0.17)	2.2 (0.24)	0.812
BUN (g/dl), mean (SD)	8.9 (4.22)	7.8 (2.29)	8.6 (3.89)	0.283	8.2 (3.44)	8.0 (3.18)	8.2 (3.37)	0.808
Cr (mg/dl), mean (SD)	0.3 (0.07)	0.3 (0.08)	0.3 (0.07)	0.180	0.3 (0.07)	0.3 (0.08)	0.3 (0.07)	0.162
CRP (mg/dl), mean (SD)	10.0 (5.21)	10.4 (4.92)	10.1 (5.12)	0.760	9.1 (5.14)	11.4 (6.82)	9.6 (5.60)	0.127
Genotype of ITPKC SNP (rs28493229 G/C)								
CC + GC: *n* (%)	22 (32.8)	3 (15.8)	25 (29.1)	0.25*	16 (24.2)	7 (36.8)	23 (27.1)	0.38*
GG: *n* (%)	45 (67.2)	16 (84.2)	61 (70.9)		48 (72.7)	12 (63.2)	60 (70.6)	
Missing	0	0	0		2	0	2	
Genotype of CASP3 SNP (rs113420705 G/A)								
AA + GA: *n* (%)	50 (74.6)	14 (73.7)	64 (74.4)	1*	42 (63.6)	13 (68.4)	55 (64.7)	1*
GG: *n* (%)	17 (25.4)	5 (26.3)	22 (25.6)		22 (33.3)	6 (31.6)	28 (32.9)	
Missing	0	0	0		2	0	2	

IVIG, intravenous immunoglobulin; CsA, ciclosporin A; CAA, coronary artery abnormality; Pre-Z-max, the maximum *Z* score of teach coronary artery before the first-line treatment; Hgb, hemoglobin; Hct, hematocrit; AST, aspartate aminotransferase; ALT, alanine aminotransferase; TB, total bilirubin; TP, total protein; Alb, albumin; Na, sodium; K, potassium; Mg, magnesium; BUN, blood urea nitrogen; Cre, creatinine; CRP, C-reactive protein; SNP, single nucleotide polymorphism.

*P*-value; no marking: equal variance two sample *t*-test.

* Fisher exact test.

** No pre-Zmax data for 2 patients (*n *= 64).

**Table 2:** Top three factors in a backward-forward stepwise logistic regression analysis for CAA risk in IVIG plus CsA group.

	OR	95% confidence interval		*P*-value
		Lower	Upper	
Pre-Z-max	17.00	4.36	66.32	<0.0001
Genotype of ITPKC SNP (rs28493229 G/C)	0.22*	0.04	1.09	0.064
Hct (%)	3.46	0.90	13.36	0.072

Pre-Z-max, the maximum *Z* score of teach coronary artery before the first-line treatment; SNP, single nucleotide polymorphism; Hct, hematocrit; OR, odds ratio.

* GC or CC were considered as the risk increasing genotypes.

### 3.2. Risk factors for resistance to initial treatment

Baseline data of the patients by treatment group and by patients' response are summarized in Table 3. In the IVIG plus CsA group, 48 patients (55.8%) responded to the first-line treatment and 38 (44.2%) were resistant. The treatment-resistant patients included 15 (17.4%) with persistent fever and 23 with relapsed fever. In the IVIG group, 48 patients (55.2%) responded to the first-line treatment and 39 (44.8%) were resistant. Those resistant to IVIG included 32 with persistent fever and 7 with relapsed fever.

**Table 3:** Difference in baseline data of KD patients who responded or not responded to the initial treatment in IVIG plus CsA and IVIG groups.

	IVIG + CsA group				IVIG group			
	Resistance (*N* = 38)	Response (*N* = 48)	Total (*N* = 86)	*P*-value	Resistance (*N* = 39)	Response (*N* = 48)	Total (*N* = 87)	*P*-value
Pre-Z-max, mean (SD)	1.5 (0.84)	1.7 (0.79)	1.6 (0.81)	0.217	1.6 (0.86)**	1.5 (0.9)**	1.5 (0.88)	0.708
Systolic pressure, mean (SD)	101.1 (12.44)	103.8 (11.42)	102.6 (11.90)	0.312	103.9 (11.14)	100.0 (12.87)	101.8 (12.21)	0.142
Diastolic pressure, mean (SD)	56.5 (9.79)	59.8 (12.30)	58.3 (11.28)	0.177	57.4 (12.10)	56.6 (12.74)	57.0 (12.38)	0.772
Heart rate, mean (SD)	149.5 (27.03)	146.7 (26.73)	147.9 (26.74)	0.628	144.8 (20.90)	143.4 (20.88)	144.0 (20.78)	0.753
WBC (10^3^/μl), mean (SD)	13.4 (5.32)	14.4 (4.19)	13.9 (4.72)	0.351	14.2 (5.16)	14.1 (4.62)	14.1 (4.84)	0.905
Neutrophil (%), mean (SD)	79.5 (11.57)	74.4 (13.00)	76.6 (12.58)	0.059	80.7 (12.98)	71.8 (15.02)	75.8 (14.76)	0.004
Lymphocyte (%), mean (SD)	14.2 (9.44)	20.1 (12.59)	17.5 (11.62)	0.018	13.9 (12.40)	22.0 (13.84)	18.3 (13.76)	0.005
Red blood cell (10^6^/μl), mean (SD)	4.4 (0.34)	4.3 (0.33)	4.3 (0.34)	0.347	4.3 (0.37)	4.4 (0.43)	4.4 (0.40)	0.300
Hgb (g/dl), mean (SD)	11.5 (0.99)	11.2 (1.19)	11.4 (1.11)	0.164	11.5 (1.26)	11.7 (1.15)	11.6 (1.20)	0.310
Hct (%), mean (SD)	34.3 (2.83)	33.5 (3.14)	33.8 (3.01)	0.246	33.8 (2.90)	34.8 (3.04)	34.4 (3.00)	0.125
Platelet (10^3^/μl), mean (SD)	278.1 (70.37)	292.8 (84.48)	286.3 (78.46)	0.393	279.4 (83.52)	306.4 (77.25)	294.3 (80.78)	0.122
AST (U/L), mean (SD)	120.2 (108.45)	98.7 (79.18)	108.2 (93.28)	0.291	174.7 (153.98)	121.6 (106.17)	145.4 (131.69)	0.061
ALT (U/L), mean (SD)	148.4 (117.22)	92.0 (84.20)	116.9 (103.41)	0.011	137.7 (114.80)	121.3 (117.74)	128.7 (116.05)	0.515
TB (mg/dl), mean (SD)	1.8 (1.45)	0.9 (0.76)	1.3 (1.21)	<0.001	1.5 (1.32)	1.1 (1.08)	1.3 (1.20)	0.096
TP (g/dl), mean (SD)	6.3 (0.59)	6.3 (0.73)	6.3 (0.67)	0.554	6.5 (0.60)	6.5 (0.63)	6.5 (0.61)	0.967
Alb (g/dl), mean (SD)	3.6 (0.42)	3.6 (0.42)	3.6 (0.41)	0.988	3.7 (0.43)	3.7 (0.44)	3.7 (0.43)	0.789
Na (mmol/L), mean (SD)	131.9 (1.99)	133.2 (2.81)	132.6 (2.54)	0.025	132.1 (3.00)	132.3 (2.74)	132.2 (2.85)	0.703
K (mmol/L), mean (SD)	4.2 (0.52)	4.1 (0.41)	4.2 (0.46)	0.607	4.2 (0.37)	4.3 (0.43)	4.2 (0.41)	0.161
Mg (mg/dl), mean (SD)	2.2 (0.24)	2.2 (0.21)	2.2 (0.22)	0.277	2.2 (0.20)	2.3 (0.27)	2.2 (0.24)	0.500
BUN (g/dl), mean (SD)	8.9 (3.09)	8.4 (4.44)	8.6 (3.89)	0.512	9.1 (3.54)	7.6 (3.21)	8.3 (3.42)	0.053
Cr (mg/dll), mean (SD)	0.3 (0.07)	0.3 (0.08)	0.3 (0.07)	0.289	0.3 (0.08)	0.3 (0.09)	0.3 (0.08)	0.159
CRP (mg/dl), mean (SD)	11.2 (5.70)	9.3 (4.50)	10.1 (5.12)	0.088	9.5 (5.82)	9.8 (5.58)	9.7 (5.65)	0.862
Body temperature before treatment, mean (SD)	39.3 (0.84)	38.9 (0.71)	39.1 (0.79)	0.018	39.4 (0.77)	38.9 (0.76)	39.1 (0.79)	0.012
Genotype of *ITPKC* SNP (rs28493229 G/C)								
CC + GC: *n* (%)	14 (36.8)	11 (22.9)	25 (29.1)	0.232*	9 (24.3)	15 (31.9)	24 (28.6)	0.477*
GG: *n* (%)	24 (63.2)	37 (77.1)	61 (70.9)		28 (75.7)	32 (68.1)	60 (71.4)	
Missing	0	0	0		2	1	3	
Genotype of *CASP3* SNP (rs113420705 G/A)								
AA + GA: *n* (%)	30 (78.9)	34 (70.8)	64 (74.4)	0.461*	27 (73.0)	29 (61.7)	56 (66.7)	0.353*
GG: *n* (%)	8 (21.1)	14 (29.2)	22 (25.6)		10 (27.0)	18 (38.3)	28 (33.3)	
Missing	0	0	0		2	1	3	

IVIG, intravenous immunoglobulin; CsA, ciclosporin A; Pre-Z-max, the maximum *Z* score of each coronary artery before the first-line treatment; Hgb, hemoglobin; Hct, hematocrit; AST, aspartate aminotransferase; ALT, alanine aminotransferase; TB, total bilirubin; TP, total protein; Alb, albumin; Na, sodium; K, potassium; Mg, magnesium; BUN, blood urea nitrogen; Cre, creatinine; CRP, C-reactive protein; SNP, single nucleotide polymorphism.

*P*-value; no marking: equal variance two sample *t*-test.

* Fisher exact test.

** Pre-Zmax information was missing for 2 resistant and 1 responsive patients.

In the IVIG plus CsA group, treatment-resistant patients had a higher ALT, higher TB, and lower Na. Meanwhile, in the IVIG group, treatment-resistant patients had a higher Neutrophil % than treatment responders (Table 3). In backward-forward stepwise multivariate logistic regression, treatment-resistant patients had a lower lymphocyte % (Ly%) and higher body temperature in both treatment groups. High serum TB levels were associated with a resistance to IVIG plus CsA, which were not associated with a response to IVIG alone (Table 4).

**Table 4:** Result of a backward-forward stepwise logistic regression analysis for resistance to the initial treatment.

	IVIG + CsA group				IVIG group			
	OR	95% confidence interval		*P*-value	OR	95% confidence interval		*P*-value
		Lower	Upper			Lower	Upper	
Body temperature before treatment	3.165	1.453	6.897	0.004	3.846	1.239	4.444	0.009
Lymphocyte (%)	1.075	1.017	1.137	0.011	1.057	1.017	1.100	0.005
TB (mg/dl)	2.342	1.297	4.219	0.005				

IVIG, intravenous immunoglobulin; CsA, ciclosporin A; OR, odds ratio; TB, total bilirubin.

## 4. Discussion

In this study, we investigated the baseline clinical characteristics associated with CAAs in KD patients treated with IVIG plus CsA. The risk factor for CAA complications was the pre-Z-max, and this factor was a strong predictor of CAAs both in the IVIG plus CsA group and in the IVIG alone group. Several reports have linked pre-Z-max to the development of CAAs (16–19). In the Post-RAISE study, which was done to evaluate the effectiveness of a combination treatment consistent of IVIG and predonisolone on reduction of CAAs, the baseline risk factors for CAA were examined in patients with a Gunma score of either ≥5 or <5. The authors determined that the risk factors for CAAs in both groups were a pre-Z-max ≥2.5, age younger than 12 months, and non-responsiveness to initial treatment, although the rankings of these three risk factors differed between previous studies (20, 21). The coronary artery findings in the early phase of KD should provide vital information for predicting coronary artery outcomes and our data re-confirm this issue.

Our univariate analysis suggested that anemia might be one of the highly important risk factors for CAAs. Baseline Hct, Hgb, and number of RBCs in the IVIG plus CsA group were shown as risk factors. Anemia has been linked to the development of CAAs (22–24). In addition, Kim et al. reported that ferritin was strongly associated with CAAs (25). Another cause of anemia might be inflammation in KD. Ferritin and haptoglobin are acute phase reactant proteins and their elevation is related to the acute phase response.

The incidence of CAAs in the IVIG plus CsA group showed a nonsignificant tendency to be lower in patients with the risk allele (C) of *ITPKC* than in those without this risk allele (odds ratio 0.22, 95% confidence interval 0.04–1.09, *p* = 0.064) (Table 2). Onouchi et al. has reported that patients with KD susceptibility alleles of *ITPKC* and/or *CASP3* were more prone to be IVIG resistance and to develop CAAs (9). While genetic studies affirmed the C allele as a risk variant for CAA, the findings in the current study didn't make conclusion about using the C allele to decide who should receive CsA. This study has inadequet power for this purpose and we need further study to evaluate this issue.

Treatment resistance in the IVIG plus CsA group is 44.2%, which was similar as that in the IVIG alone group (44.8%). This fact is unlikely to motivate physicians to select IVIG plus CsA treatment for high-risk KD. In this study, we could not find positive factors for choosing the IVIG plus CsA treatment compared to the IVIG alone. Resistance to first-line treatment, both in IVIG plus CsA group and IVIG alone group, revealed that body temperature before treatment and percent of lymphocytes were significantly related to resistance. Body temperature before treatment is a simple but important parameter that can be readily obtained worldwide. In a previous study, body temperature before treatment has been reported to be a good predictor of IVIG resistance (26).

Serum TB level was an independent predictor of first-line therapy resistance in the IVIG plus CsA group but not in the IVIG alone group. Because CsA is a fat-soluble drug, the absorption of CsA from the gastrointestinal tract may decrease in patients with cholestasis. However, the trough levels of CsA showed no significant differences between patients with and without a response to IVIG plus CsA (data not shown). Baseline TB levels have also been reported to be a risk factor for resistance to intensified IVIG plus prednisolone treatment (20). Although not significant, patients who were resistant to the standard IVIG treatment tended to have higher TB than those responded to the treatment (Table 3). Thus, we speculate that high TB may reflect the predominance of factors that are difficult to resolve with the addition of CsA or prednisolone in the complex and heterogeneous pathogenesis of refractory KD, and that the relative contribution of these factors may have been accentuated by the administration of CsA or prednisolone.

It is vital to select the most appropriate first-line treatment for KD patients to prevent CAAs. However, we could not confirm any significant independent factor for selecting between IVIG plus CsA and IVIG alone in this study. Since this study is a sub-analysis, we know that there is a problem with the statistical power, which is a limitation of this study. In addition, the research was conducted only on Japanese subjects, and the data cannot be used universally.

In conclusion, Pre-Z-max of coronary arteries was a risk factor significantly associated with CAAs in patients who are high risk for IVIG resistance and received IVIG plus CsA treatment. Baseline serum total bilirubin level was a risk factor associated with resistance to IVIG plus CsA.

## Data Availability

The raw data supporting the conclusions of this article will be made available by the authors, without undue reservation.

## References

[1] Kawasaki T. Acute febrile mucocutaneous syndrome with lymphoid involvement with specific desquamation of the fingers and toes in children. Arerugi. (1967) 16:178–222. 6062087

[2] Gordon JB Kahn AM Burns JC. When children with Kawasaki disease grow up: myocardial complications in adulthood. J Am Coll Cardiol. (2009) 54:1911–20. 10.1016/j.jacc.2009.04.102 19909870 PMC2870533

[3] McCrindle BW Rowley AH Newburger JW Burns JC Bolger AF Gewitz M Diagnosis, treatment, and long-term management of Kawasaki disease: a scientific statement for health professionals from the American heart association. Circulation. (2017) 135:e927–99. 10.1161/CIR.0000000000000484 28356445

[4] Newburger JW Takahashi M Beiser AS Burns JC Bastian J Chung KJ A single intravenous infusion of gamma globulin as compared with four infusions in the treatment of acute Kawasaki syndrome. N Engl J Med. (1991) 324:1633–9. 10.1056/NEJM199106063242305 1709446

[5] Hamada H Suzuki H Onouchi Y Ebata R Terai M Fuse S Efficacy of primary treatment with immunoglobulin plus ciclosporin for prevention of coronary artery abnormalities in patients with Kawasaki disease predicted to be at increased risk of non-response to intravenous immunoglobulin (KAICA): a randomised controlled, open-label, blinded-endpoints, phase 3 trial. Lancet. (2019) 393(10176):1128–37. 10.1016/S0140-6736(18)32003-8 30853151

[6] Kobayashi T Saji T Otani T Takeuchi K Nakamura T Arakawa H Efficacy of immunoglobulin plus prednisolone for prevention of coronary artery abnormalities in severe Kawasaki disease (RAISE study): a randomised, open-label, blinded-endpoints trial. Lancet. (2012) 379(9826):1613–20. 10.1016/S0140-6736(11)61930-2 22405251

[7] Tremoulet AH Jain S Jaggi P Jimenez-Fernandez S Pancheri JM Sun X Infliximab for intensification of primary therapy for Kawasaki disease: a phase 3 randomised, double-blind, placebo-controlled trial. Lancet. (2014) 383(9930):1731–8. 10.1016/S0140-6736(13)62298-9 24572997

[8] Onouchi Y Gunji T Burns JC Shimizu C Newburger JW Yashiro M ITPKC functional polymorphism associated with Kawasaki disease susceptibility and formation of coronary artery aneurysms. Nat Genet. (2008) 40:35–42. 10.1038/ng.2007.59 18084290 PMC2876982

[9] Onouchi Y Suzuki Y Suzuki H Terai M Yasukawa K Hamada H ITPKC and CASP3 polymorphisms and risks for IVIG unresponsiveness and coronary artery lesion formation in Kawasaki disease. Pharmacogenomics J. (2013) 13:52–9. 10.1038/tpj.2011.45 21987091

[10] Kobayashi T Inoue Y Takeuchi K Okada Y Tamura K Tomomasa T Prediction of intravenous immunoglobulin unresponsiveness in patients with Kawasaki disease. Circulation. (2006) 113:2606–12. 10.1161/CIRCULATIONAHA.105.592865 16735679

[11] Miura M Ayusawa M Fukazawa R Hamada H Ikeda S Ito S The guidelines on acute stage Kawasaki disease treatment. Pediatr Cardiol Cardiac Surg. (2020) 36(S1):S1.1–.29.

[12] Yang JC Jain S Capparelli EV Best BM Son MB Baker A Anakinra treatment in patients with acute Kawasaki disease with coronary artery aneurysms: a phase I/IIa trial. J Pediatr. (2022) 243:173–80. 10.1016/j.jpeds.2021.12.035 34953816

[13] Kone-Paut I Tellier S Belot A Brochard K Guitton C Marie I Phase II open-label study of anakinra in intravenous immunoglobulin-resistant Kawasaki disease. Arthritis Rheumatol. (2021) 73:151–61. 10.1002/art.41481 32779863

[14] Ayusawa M Sonobe T Uemura S Ogawa S Nakamura Y Kiyosawa N Revision of diagnostic guidelines for Kawasaki disease (the 5th revised edition). Pediatr Int. (2005) 47:232–4. 10.1111/j.1442-200x.2005.02033.x 15771703

[15] Kobayashi T Fuse S Sakamoto N Mikami M Ogawa S Hamaoka K A new *Z* score curve of the coronary arterial internal diameter using the lambda-mu-sigma method in a pediatric population. J Am Soc Echocardiogr. (2016) 29:794–801. 10.1016/j.echo.2016.03.017 27288089

[16] Fuse S Mori T Kuroiwa Y Hirakawa S. On what day of, illness does the dilatation of coronary arteries in patients with, Kawasaki disease begin? Circ J. (2017) 82:247–50. 10.1253/circj.CJ-17-0046 28845030

[17] Suzuki T Kakimoto N Tsuchihashi T Suenaga T Takeuchi T Shibuta S Z-score is a possible predictor of the risk of coronary artery lesion development in patients with Kawasaki disease in Japan. Eur J Pediatr. (2021) 180:2797–805. 10.1007/s00431-021-04006-1 33763715

[18] Son MBF Gauvreau K Tremoulet AH Lo M Baker AL de Ferranti S Risk model development and validation for prediction of coronary artery aneurysms in Kawasaki disease in a north American population. J Am Heart Assoc. (2019) 8:e011319. 10.1161/JAHA.118.011319 31130036 PMC6585355

[19] Crystal MA Manlholt C Yeung RSM Smallhorn JF McCrindle BW. Coronary artery dilation after Kawasaki disease for children within the normal range. Int J Cardiol. (2009) 136:27–31. 10.1016/j.ijcard.2008.04.019 18619690

[20] Miyata K Miura M Kaneko T Morikawa Y Sakakibara H Matsushima T Risk factors of coronary artery abnormalities and resistance to intravenous immunoglobulin plus corticosteroid therapy in severe Kawasaki disease: an analysis of post RAISE. Circ Cardiovasc Qual Outcomes. (2021) 14:e007191. 10.1161/CIRCOUTCOMES.120.007191 33541111

[21] Iio K Morikawa Y Miyata K Kaneko T Misawa M Yamagishi H Risk factors of coronary artery aneurysms in Kawasaki disease with a low risk of intravenous immunoglobulin resistance: an analysis of post RAISE. J Pediatr. (2022) 240:158–63. 10.1016/j.jpeds.2021.08.065 34461064

[22] Kuo HC Yang YL Chuang JH Tiao MM Yu HR Huang LT Inflammation-induced hepcidin is associated with the development of anemia and coronary artery lesions in Kawasaki disease. J Clin Immunol. (2012) 32:746–52. 10.1007/s10875-012-9668-1 22392047

[23] Huang YH Kuo HC Huang FC Yu HR Hsieh KS Yang YL Hepcidin-induced iron deficiency is related to transient anemia and hypoferremia in Kawasaki disease patients. Int J Mol Sci. (2016) 17:715. 10.3390/ijms17050715 27187366 PMC4881537

[24] Huang YH Kuo HC. Anemia in Kawasaki disease: hepcidin as a potential biomarker. Int J Mol Sci. (2017) 18:820. 10.3390/ijms18040820 28417923 PMC5412404

[25] Kim S Eun LY. Iron deficiency anemia as a predictor of coronary artery abnormalities in Kawasaki disease. Korean J Pediatr. (2019) 62:301–6. 10.3345/kjp.2018.06905 30744317 PMC6702116

[26] Gámez-González LB Hamada H Cisneros CM Honda T Yasukawa K Takanashi J. Vital signs as predictor factors of intravenous immunoglobulin resistance in patients with Kawasaki disease. Clin Pediatr. (2018) 57:1148–53. 10.1177/0009922818759320 29486579

